# Enhancing HIV Testing and Treatment among Men Who Have Sex with Men in China: A Pilot Model with Two-Rapid Tests, Single Blood Draw Session, and Intensified Case Management in Six Cities in 2013

**DOI:** 10.1371/journal.pone.0166812

**Published:** 2016-12-01

**Authors:** Dapeng Zhang, Hongyan Lu, Minghua Zhuang, Guohui Wu, Hongjing Yan, Jun Xu, Xiaoli Wei, Chengmei Li, Sining Meng, Xiaojing Fu, Jinlei Qi, Peng Wang, Mei Luo, Min Dai, Ray Yip, Jiangping Sun, Zunyou Wu

**Affiliations:** 1 National Center for AIDS/STD Control and Prevention, Chinese Center for Disease Control and Prevention, Beijing, China; 2 Beijing Center for Diseases Control and Prevention, Beijing, China; 3 Shanghai Municipal Center for Disease Control and Prevention, Shanghai, China; 4 Chongqing Center for Disease Control and Prevention, Chongqing, China; 5 Jiangsu Provincial Center for Disease Control and Prevention, Nanjing, China; 6 Wuhan Centers for Disease Prevention and Control, Wuhan, China; 7 Xi’an Center for Disease Control and Prevention, Xi’an, China; 8 Chinese Preventive Medicine Association, Beijing, China; 9 Chinese Association of STD & AIDS Prevention and Control, Beijing, China; 10 Bill & Melinda Gates Foundation China Office, Beijing, China; University of New South Wales, AUSTRALIA

## Abstract

**Objectives:**

To explore models to improve HIV testing, linkage to care and treatment among men who have sex with men (MSM) in cooperation with community-based organizations (CBOs) in China.

**Methods:**

We introduced a new model for HIV testing services targeting MSM in six cities in 2013.These models introduced provision of rapid HIV testing by CBO staff and streamlined processes for HIV screening, confirmation of initial reactive screening results, and linkage to care among diagnosed people. We monitored attrition along each step of the continuum of care from screening to treatment and compared program performance between 2012 and 2013. According to the providers of two rapid tests (HIV screening), four different services delivery models were examined in 2013: Model A = first screen at CDC, second at CDC (Model A = CDC+CDC), Model B = first and second screens at CBOs (Model B = CBO+CBO), Model C = first screen at CBO, second at Hospital (Model C = CBO+Hosp), and Model D = first screen at CBO, second at CDC (Model D = CBO+CDC). Logistic regressions were performed to assess advantages of different screening models of case finding and case management.

**Results:**

Compared to 2012, the number of HIV screening tests performed for MSM increased 35.8% in 2013 (72,577 in 2013 vs. 53,455 in 2012). We observed a 5.6% increase in proportion of cases screened reactive receiving HIV confirmatory tests (93.9% in 2013 vs. 89.2% in 2012, χ^2^ = 48.52, p<0.001) and 65% reduction in loss to CD4 cell count tests (15% in 2013 vs. 43% in 2012, χ^2^ = 628.85, p<0.001). Regarding linkage to care and treatment, the 2013 pilot showed that the Model D had the highest rate of loss between screening reactive and confirmatory test among the four models, with 18.1% fewer receiving a second screening test and a further 5.9% loss among those receiving HIV confirmatory tests. The Model B and the Model C showed lower losses (0.8% and 1.3%) for newly diagnosed HIV positives receiving CD4 cell count tests, and higher rates of HIV positives referred to designated ART hospitals (88.0% and 93.3%) than the Model A and Model D (4.6% and 5.7% for CD4 cell count test, and 68.9% and 64.4% for referring to designated ART hospitals). The proportion of cases where the screening test was reactive that were commenced on ART was highest in Model C; 52.8% of cases commenced on ART compared to 38.9%, 34.2% and 21.1% in Models A, B and D respectively. Using Model A as a reference group, the multivariate logistic regression results also showed the advantages of Models B, C and D, which increased CD4 cell count test, referral to designated ART hospitals and initiation of ART, when controlling for program city and other factors.

**Conclusions:**

This study has demonstrated that involvement of CBOs in HIV rapid testing provision, streamlining testing and care procedures and early hospital case management can improve testing, linkage to, and retention in care and treatment among MSM in China.

## Introduction

Recent studies, including prospective cohort studies [1, 2], randomized controlled trials [3] and mathematical modeling [4–8], have provided strong evidence showing that HIV transmission via heterosexual and homosexual contact can be reduced or prevented by effective antiretroviral therapy (ART). Given its dual benefits of both improving the quality of life of people living with HIV/AIDS (PLHIV) and reducing the spread of HIV, ART has become the cornerstone of an effective response to the HIV/AIDS epidemic. However, because expansion of ART relies on early diagnosis of PLHIV, many countries have explored and developed a “Test-and-Treat” public health approach [9–13].

After 30 years, China’s HIV epidemic is still increasing, with nearly 40% of PLHIV unaware of their HIV infection status. The rapidly escalating epidemic among men who have sex with men (MSM) is of particular concern [14]. China has adopted strategies for HIV prevention among MSM that seek to expand HIV testing and counseling (HTC) as a means to promote HIV case finding and ART uptake [15–18]. This commitment is documented in the Chinese State Council’s Five Year Action Plan for HIV Prevention (2011–2015) [19].

Supported by the Bill & Melinda Gates Foundation, a cooperative program for HIV prevention among MSM in key urban centers within China was established in 2008. The program addressed HIV testing and linkage to care and treatment by promoting cooperation between community-based organizations (CBOs), Centers for Disease Control and Prevention (CDCs), and hospitals [15, 20]. Within this model, CBOs were responsible for HIV testing mobilization among MSM and referring MSM to local CDCs for HIV services [20, 21]. Because only approved medical institutions can perform HIV testing in China, HIV testing was mainly performed by CDC staff, either in CDC offices or in collaboration with CBOs at CBO offices or gay venues and meeting places, such as bars, saunas and public parks. The program demonstrated the feasibility and effectiveness of enhancing cooperation across public health departments, medical institutions, and CBOs. However, two key challenges remained [10, 13, 22]. Firstly, HIV testing coverage was still below 20% in 2012, and nearly 50% of MSM tested in 2012 had never been tested previously (unpublished program data). Secondly, attrition was still significant, with 16% of MSM screened HIV-reactive not receiving HIV confirmatory testing and 20% of newly-identified cases not receiving CD4 testing [15].

The high degree of attrition may be attributable to systemic barriers in the Chinese standard of care protocol for diagnosing HIV infection and linking patients to medical care [15]. In order to make a diagnosis, three separate patient-provider encounters are required, with blood drawn at different clinics for initial screening, second screening, and confirmatory testing. If the initial HIV screening test is reactive, the patient has to be contacted and asked to return to the same or another clinic for a second time to have blood drawn and the second screening test is performed. If the second screening test is reactive, the second blood sample is then sent to centralized public health laboratories at CDC facilities that serve an entire city or district. There, blood samples for HIV confirmatory testing are batched for processing, and it generally takes at least 10 to 15 days for results to return to the originating clinic. After results arrive, patients must again be located and notified of their test results.

Persons diagnosed with HIV infection are then asked to visit the clinic for the third time to be tested for CD4 cell count. CD4 testing is also performed at CDC public health laboratories in batches, again usually taking 10 to 15 days for results. Once CD4 cell count test results are available, patients must be located yet again to inform them of their CD4 cell count test results and to ask that they return to a hospital or clinic for evaluation for ART eligibility.

Although it has been shown that rapid HIV testing can streamline the testing process and shorten the time between testing and treatment [23–26], traditional enzyme immunoassay (EIA) methods are still the primary HIV screening method employed in China. One possible reason may be that rapid tests are more time consuming than batch-processed EIAs when the number of samples is large, making CDC laboratory staff with constant heavy workloads more likely to favor traditional EIA methods [27]. However, rapid HIV testing has also been shown to be particularly suited to community-based testing [23–26]. Furthermore, CBO staff, with more flexibility and greater knowledge of the MSM community, may be better positioned for case finding than CDC workers, particularly when CBOs are equipped with rapid tests. This format may be especially beneficial for the many MSM who are not comfortable with traditional public health clinics [25, 26, 28]. Nevertheless, this approach is limited by China’s HIV testing policy—CBO staff often lack the medical qualifications required to legally conduct HIV testing. Local CDCs, as governmental institutions, often also have legal concerns about supporting HIV rapid testing services at CBOs. This further limits the realization of the potential contributions CBOs could make to HIV case finding.

The issues of poor testing uptake and high attrition of HIV-reactive MSM along the continuum care is not unique to China, but rather, is a global challenge that many countries still face [9, 10, 12, 29]. Operational models providing evidence of a beneficial role for CBO in HIV testing and case management are urgently needed for China to respond to the burgeoning HIV epidemic among MSM. Therefore, the aim of the present study was to assess the effectiveness of a pilot model consisting of two-rapid tests by CBOs, single blood draw session, and intensified case management with the support of CBOs among MSM in six Chinese cities.

## Materials and Methods

The study was conducted in six cities (Beijing, Chongqing, Shanghai, Nanjing, Wuhan, and Xi’an) in China from January to December in 2013. The six cities were selected from the original 15 program sites (14 cities and Hainan Province) included in the cooperative program for HIV prevention among MSM supported by the Bill & Melinda Gates Foundation. City selection was based on strong working relationships between local governments and local CBOs, relatively high HIV prevalence among MSM, and local CBOs with demonstrated capacity and previous experience working on HIV prevention and care programs.

### Program interventions

The core strategy of the 2013 pilot was the same as the previous China-Gates HIV Program, which was based on the “test-and-treat” public health approach [20]. To address the challenges of the previous program, three key interventions were implemented for the 2013 pilot:

**Intervention I**: (a) to use HIV rapid testing instead of traditional EIA methods for initial screening, and (b) to encourage CBOs to conduct rapid HIV testing among MSM in addition to community mobilization for HIV testing.

**Intervention II**: to accelerate referral procedures and expedite linkage to care using a “streamlined service model” that (a) refers MSM with reactive screening results to just one clinic (CDCs or ART hospitals) where blood samples are drawn in one clinic visit (instead of the traditional two additional visits) for confirmatory, CD4 and viral load (VL) tests; and (b) provides intensified case management (post-test counseling, confirmatory, CD4, and VL testing, ART eligibility screening and counseling, and referral for ART initiation) within four weeks of being screened reactive at the same clinic where blood was drawn.

**Intervention III**: to refer HIV-reactive MSM to ART hospitals (instead of CDCs) for case management immediately after screening. CBOs provide support for case referral and work together with CDCs and hospitals to provide care and support for newly-diagnosed cases.

### Service delivery models

**Service delivery model in 2012:** CBOs, mostly as recruiters, were responsible for conducting health education and behavioral intervention among MSM and mobilizing them to receive HIV tests. HIV testing itself was provided by CDC staff either in local CDCs or at testing points such as bars, saunas and public parks or CBO offices. Approximately 50% of screening tests used were rapid tests and approximately 50% were traditional EIAs. Referral mechanisms were established between CBOs and local CDCs to ensure MSM who screened reactive would receive HIV confirmatory and CD4 testing at CDCs. Eligible individuals were referred to designated hospitals for ART.

**Fig 1:**
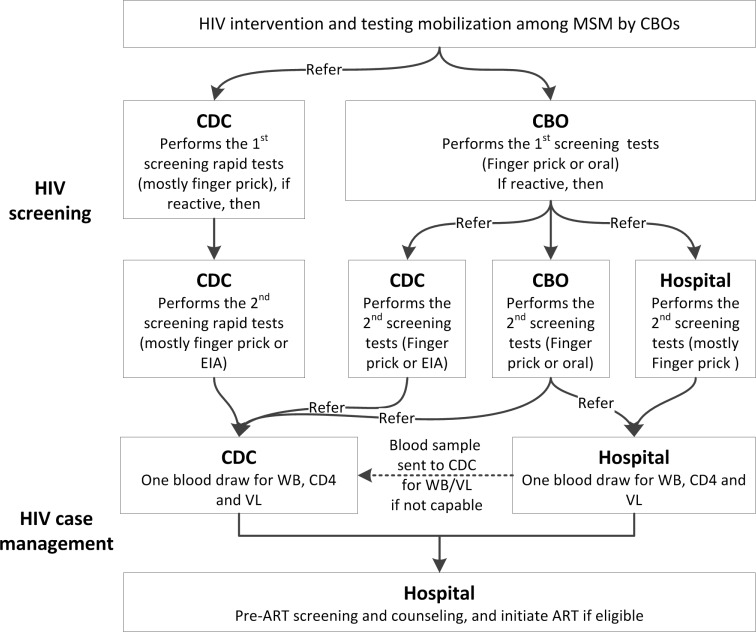
Service delivery models for HIV testing and case management in 2013

**Screening service delivery models in 2013:** Four screening service delivery models (**Fig 1**) were developed and are described as follows:

- **Model A** (CDC+CDC): Both first and second screening tests were done by CDC staff, and the HIV-reactive MSM was followed with intensified case management Model I described below.
- **Model B** (CBO+CBO): Both first and second screening tests were rapid tests performed by CBO staff, and the HIV-reactive MSM was followed with intensified case management Model I or Model II described below.
- **Model C** (CBO+Hosp): CBO staff performed the first rapid test. Those screened reactive were referred by CBOs to ART hospitals for a second rapid test, and the HIV-reactive MSM was followed with intensified case management Model II described below. In this model, most CBOs were located in the hospitals working closely with clinicians.
- **Model D** (CBO+CDC): CBO staff performed the first rapid test. Those screened reactive were referred by CBOs to local CDCs for a second screening test, and the HIV-reactive MSM was followed with intensified case management Model I described below.

Case management for those who screened HIV-reactive was conducted in the format of two different models:

- **Model I**: individuals who screened reactive were referred to CDCs to receive HIV confirmatory tests, CD4 cell count and VL tests, and then were referred to designated hospitals for counseling, to assess ART eligibility, and to initiate ART initiated if deemed eligible.
- **Model II**: individuals who screened reactive were referred to designated hospitals to receive HIV confirmatory test (WB), CD4 cell count and VL tests, as well as counseling, assessment of ART eligibility, and ART initiation if eligible.

### Testing

HIV rapid testing kits were procured at the national level and distributed to the six project cities. About 60% were the Determine HIV-1/2® Rapid Test (Abbott Laboratories, Japan), and 40% were the Oral Mucosal Transudate HIV-1/2 Rapid Test (of them, 50% were from the Marro Bio-Pharmaceutical Co, Beijing, China; 25% from the Xiehe Bio-technical Co, Chengdu, China[30]; and 25% from Wandfo Bio-technical Co, Guangzhou, China[31]). All these test kits are approved by China State Food and Drug Administration (SFDA), with a sensitivity of >97% and specificity of >97%, and results available within 30 minutes [32]. A small proportion of tests were performed using EIA, and the type of EIA kits varied based on local situations. HIV confirmatory testing (by Western blot), CD4 cell count testing and VL testing were done by existing public health laboratories in the six study cities.

### Data collection

MSM who screened positive for HIV were interviewed using a standard questionnaire. The questionnaire included two parts: HIV testing and case management. CBOs were responsible for conducting interviews to fill in the first part of the questionnaire (testing), which included information on socio-demographic characteristics, sexual behaviors, condom use, and HTC history to HIV screening results. Local CDCs or ART hospitals were responsible for conducting interviews to fill in the second part (case management), which included information on timing, results and providers of confirmatory, CD4 count, and VL tests, and subsequent ART initiation. Collected information was entered into the program’s web-based data collection tool and each questionnaire was assigned a unique code. This unique code was an anonymous identifier assigned to the client to remove the need to have the client’s citizen identification card number.

### Data analysis

Data analysis focused on three areas. Firstly, the attrition along each step of the continuum of HIV care from screening to treatment was analyzed. Secondly, a comparison of program performance between 2012 and 2013 was presented to assess changes in testing and linkage to care, focusing on HIV confirmatory testing, CD4 cell count testing, and ART initiation among MSM who screened reactive. Pearson Chi square test was used to test the significance of differences between the two project periods. Thirdly, differences in the impact of testing and treatment between the four screening service delivery models were analyzed using bivariate and multivariate methods. Four logistic regressions were performed to assess case finding and case management advantages of different screening models (**Table 1**). Differences between the four screening service delivery models in the time period between each key step from screening to treatment were also analyzed using Kruskal-Wallis equality-of- populations rank testing. To improve the comparability of participants in the four screening service delivery models, 168 MSM (0.2% of the total sample), whose first HIV screening was conducted by EIA, and 191 MSM (0.3% of the total sample), whose first tests were performed by institutions other than CDCs or CBOs were excluded from the comparison analysis. The differences between CDCs and ART hospitals as case management providers were also analyzed. However, we found that, among cases screened reactive by the CBO+CBO model, nearly 90% were referred to CDCs for HIV case management (only 10% were referred to hospitals). Therefore, to simplify the analysis, we only focused on the analysis of four models based on the HIV screening process. In 2013, a total of 72,577 HIV screening tests were performed among MSM.

**Table 1:** Variable assignments of logistic regression analysis.

logistic regression	Dependent variables	Independent variable	Controlling variables
Model 1 (n = 3089)	Whether receiving WB confirmatory test for HIV screened reactive (1 = WB tested, 0 = Otherwise)	Service delivery models: Dummy coded with the CDC+CDC group as the reference group. (CDC+CDC = 1 if both rapid tests done by CDC, otherwise = 0; CBO+CBO = 1 if both rapid tests done by CBO, otherwise = 0; CBO+Hosp = 1 if first done by CBO and second by hospital, otherwise = 0; CBO+CDC = 1 if first done by CBO and second by CDC, otherwise = 0).	Socio demographics: age, education, marriage status, number of sexual partners, HIV testing history, recruitment channel, and program city.
Model 2 (n = 2706)	Whether receiving CD4 cell count test for newly identified HIV positives (1 = CD4 tested, 0 = Otherwise)		
Model 3 (n = 2706)	Whether being referred to the designated ART hospitals for newly identified HIV positives (1 = Referred to hospital case management, 0 = Otherwise)		
Model 4 (n = 2052)	Whether initiated ART for newly identified HIV positives (1 = ART initiated, 0 = Otherwise)		

### Ethics statement

This study introduces no new testing procedures or treatment interventions. There is no increased risk with HIV testing, with the use of HIV test methods already in current practice. All HIV consent and testing procedures followed relevant Chinese national guidelines. Written informed consent was not obtained because it may result in refusal to participate by some patients, and allowing for withdrawal of study consent could result in the loss of some study data, which would compromise the validity of study results. A participant handout describing the study, HIV testing, and data collection procedures was provided to all participants. Verbal consent was obtained prior to participants receiving HIV screening and subsequent tests. Participants were allowed to opt out of having their data used for the study. Receipt of participant handout without opting out was documented and was treated as consent for participating in the program. The study protocol and consent procedure were reviewed and approved by the Institutional Review Board of the National Center for AIDS/STD Control and Prevention, Chinese Center for Disease Control and Prevention.

## Results

### Attrition from HIV screening to ART initiation

**Fig 2:**
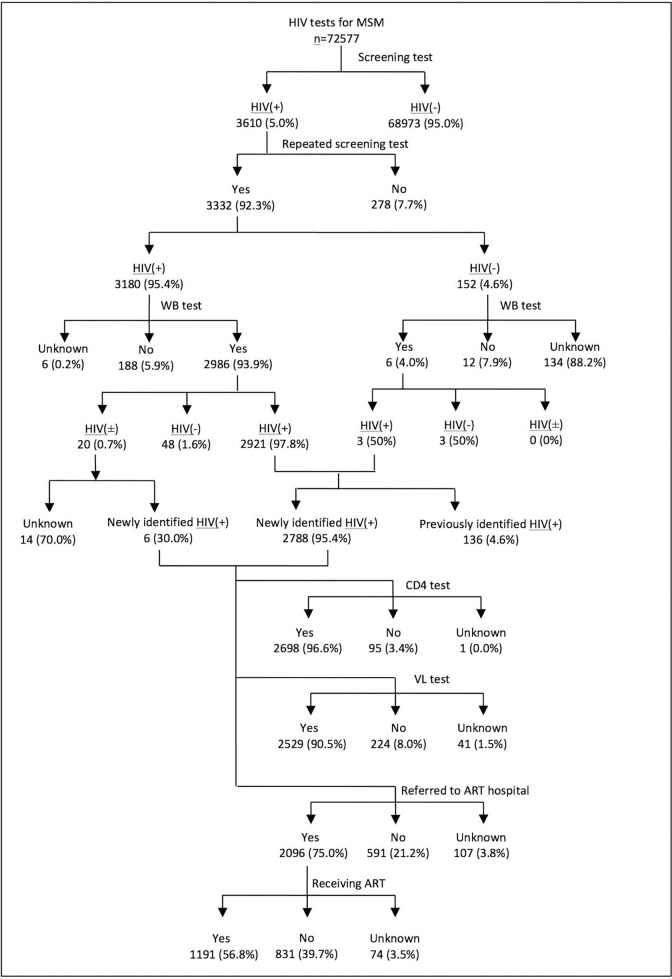
Attrition of HIV continuum of care from screening to ART initiation in the six project cities in China, 2013

Attrition along the continuum of care from initial HIV screening to ART mainly occurred in four steps (**Fig 2**): 7.7% (278/3,610) of those screened reactive with the first screening test did not receive second screening tests; 5.9% (188/3,180) of those who screened reactive a second time did not receive HIV confirmatory tests; 21.2% (591/2,794) of the newly-diagnosed HIV positive MSM were not successfully referred to ART hospitals; and 39.7% (831/2,096) of those who were referred to ART hospitals did not initiate ART by the end of 2013.

### Improvements in linkage to care and treatment

Compared with 2012 the number of HIV screening tests performed for MSM increased by 35.8%, with 72,577 tests (**S1 Table**) performed in 2013 (accounting for 25.0% of the estimated MSM population in the six cities) compared to 53,455 tests performed in 2012 (18.4% of the esitmated MSM population; **Table 2**). The proportion of HIV-reactive MSM obtaining confirmatory tests increased from 89.2% in 2012 to 93.9% in 2013 (χ^2^ = 48.52, p<0.001). The proportion of newly-identified HIV-positive MSM receiving CD4 cell count tests increased from 77.2% in 2012 to 96.6% in 2013. Overall, if the number of screened reactives is used as the denominator, the loss to follow-up in the process up to CD4 cell count test for screened reactives was reduced by 64.7% (43.0% in 2012 vs. 15.2% in 2013, χ^2^ = 628.85, p<0.001). The proportion of cases screened reactive in 2013 was lower than that in 2012 (4.4% vs. 6.8%, χ^2^ = 375.14, p<0.001) and further led to lower efficiency in detecting newly-diangosed cases (3.9% or 2,794 diagnosed cases/72,577screening tests in 2013 vs. 5.1% or 2,717 diagnosed cases/53,455 tests in 2012; χ^2^ = 111.93, p<0.001).

**Table 2:** Attrition of MSM who screened reactive along the HIV continuum of care, from initial screening to treatment in China: comparing 2012 to 2013.

Indicators	2012	2013	χ^2^	P value
Estimated MSM population size in 2011	289,940	289,940	-	-
HIV tests among MSM (% of estimated MSM)	53,455 (18.4%)	72,577 (25.0%)	-	-
Screened reactive (% of screened)	3,681 (6.8%)	3,180 (4.4%)	375.14	0.000
Informed of reactive screening results (% of screening reactive)	3,667 (99.6%)	3,180 (100%)	-	0.000^3^
Received confirmatory tests (% of screened reactive)	3,282 (89.2%)	2,986 (93.9%)	48.52	0.000
Confirmed positive (% of those confirmed—Positive predictive value)	3,025 (92.2%)	2,921 (97.8%)	102.55	0.000
Newly reported infections (% of confirmed positives)^1^	2,717 (89.8%)	2,794 (95.7%)	74.59	0.000
Efficiency of identifying new infections	5.1%	3.9%	111.93	0.000
Received CD4 Testing (% of newly-reported)	2,098 (77.2%)	2,698 (96.6%)	456.63	0.000
CD4 count < 350 cells/μl (% of received CD4 testing)	749 (35.7%)	1,351 (50.1%)	99.06	0.000
Initiated ART (% of newly-reported infections^)^ ^2^	-	1,191 (42.6%)	-	-

^1^Removed previously confirmed positive (against the National AIDS Case Reporting System).

^2^ART initiation data was not available in 2012.

^3^Fisher’s exact test.

### Differences between service delivery models in testing, linkage to care, and treatment

After classifying the data based on the four models (**Table 3**), Model A (first screening test by CDC) showed a higher proportion of cases screened reactive than Model B, C and D in which the first screening test was performed by CBOs (11.1% vs. 3.9%, p<0.001). After stratifying by cities, HIV prevalence was higher among MSM screened by CDCs in three cities (Chongqing: 21.5% [267/1243] vs. 6.3% [413/6559], p<0.001; Nanjing: 6.9% [128/1845] vs. 2.0% [153/7542], p<0.001; Shanghai: 10.3% [723/6998] vs. 4.1% [98/2369], p<0.001) The other three cities were not included in this analysis because most first screening tests there were conducted by CBOs.

**Table 3:** Sample characteristics of MSM screened by 4 service delivery models.

Model	A = CDC+CDC	B = CBO+CBO	C = CBO+Hosp	D = CBO+CDC
**First screening test performed by:**	**CDC (n = 10,133)**	**CBO (n = 62,043)**		
City where screening took place				
Beijing	2 (0.0)	26,103 (42.1)		
Chongqing	1, 243 (12.3)	6,559 (10.6)		
Nanjing	1,845 (18.2)	7,542 (12.2)		
Shanghai	6,998 (69.1)	2,369 (3.8)		
Wuhan	5 (0.1)	10,378 (16.7)		
Xi’an	40 (0.4)	9,902 (14.7)		
Total	10,133 (100.0)	62,043 (100.0)		
Results of first screening				
Reactive	1,122 (11.1)	2,392 (3.9)		
Non-reactive	9,011 (88.9)	59,651 (96.1)		
Total	10,133 (100.0)	62,043 (100.0)		
**Second screening test performed by:**	**CDC (n = 1,122)**	**CBO (n = 870)**	**HOSP (n = 324)**	**CDC (n = 1,198)**
Received second screening test (among those screened reactive by first screening test) *				
Yes	1,100 (98.0)	835 (96.0)	324 (100.0)	981 (81.9)
No	22 (2.0)	35 (4.0)	0 (0.0)	217 (18.1)
Total	1,122 (100.0)	870 (100.0)	324 (100.0)	1,198(100.0)
Results of second screening test *				
Reactive	1,094 (99.5)	828 (99.2)	316 (97.5)	851 (86.7)
Non-reactive	6 (0.5)	7 (0.8)	8 (2.5)	130 (13.3)
Total	1,100(100.0)	835 (100.0)	324 (100.0)	981 (100.0)
Days between 1^st^ and 2^nd^ screening test (day)				
Mean ± SD ^#^	0.94±5.3	0.70±4.7	0.55±3.8	3.3±12.4
<1	863 (78.6)	748 (89.6)	297 (91.7)	337 (34.5)
1	105 (9.6)	23 (2.8)	16 (4.9)	184 (18.8)
2–7	91 (8.3)	36 (4.3)	4 (1.3)	357 (36.4)
≥8	32 (2.9)	24 (2.9)	7 (2.2)	94 (9.6)
Unknown	9 (0.8)	4 (0.5)	0 (0.0)	9 (0.9)
Total	1100	835	324	981
Received WB confirmatory test *				
Yes	990 (90.5)	807 (97.5)	308 (97.4)	799 (93.9)
No	104 (9.5)	21 (2.5)	4 (1.3)	50 (5.9)
Unknown	0 (0.0)	0 (0.0)	4 (1.3)	2 (0.2)
Total	1,094 (100.0)	828 (100)	316 (100.0)	851 (100.0)
Results of WB confirmatory test				
HIV(+)	977 (98.7)	795 (98.5)	299 (97.1)	769 (96.3)
HIV(-)	2 (0.2)	7 (0.9)	9 (2.9)	27 (3.4)
Ambiguous	11 (1.1)	5 (0.6)	0 (0.0)	3 (0.4)
Total	990 (100.0)	807 (100.0)	308 (100.0)	799 (100.0)
Newly diagnosed HIV positives				
Yes	927 (94.9)	767 (96.5)	298 (99.7)	714 (92.9)
No	50 (5.1)	28 (3.5)	1 (0.3)	55 (7.2)
Total	977 (100.0)	795 (100.0)	299 (100.0)	769 (100.0)
Days between 2^nd^ screening test and WB confirmatory test (day)				
Mean ± SD^#^	6.1±10.9	14.3±16.5	12.6±16.5	8.5±14.6
<1	163 (16.5)	21 (2.6)	26 (8.4)	129 (16.2)
1	144 (14.6)	23 (2.9)	5 (1.6)	89 (11.1)
2–7	449 (43.4)	299 (37.1)	68 (22.1)	311 (38.9)
≥8	203 (20.5)	443 (54.9)	200 (64.9)	262 (32.8)
Unknown	31 (3.1)	21 (2.6)	9 (2.9)	8 (1.0)
Total	990	807	308	799
Received CD4 cell count test for newly diagnosed HIV positives *				
Yes	884 (95.4)	761 (99.2)	294 (98.7)	672 (94.1)
No	43 (4.6)	6 (0.8)	4 (1.3)	41 (5.7)
Unknown	0 (0.0)	0 (0.0)	0 (0.0)	1 (0.1)
Total	927 (100.0)	767 (100.0)	298 (100.0)	714 (100.0)
Days between 2^nd^ screening test and CD4 cell count test (day)				
Mean ± SD^#^	12.1±19.7	19.9±29.1	3.2±10.6	16.2±27.6
<1	169 (19.1)	64 (8.4)	103 (35.0)	107 (15.9)
1	122 (13.8)	71 (9.3)	89 (30.3)	82 (12.2)
2–7	216 (24.4)	213 (28.0)	65 (22.1)	149 (22.2)
≥8	367 (41.5)	408 (53.6)	34 (11.6)	333 (49.6)
Unknown	10 (1.1)	5 (0.7)	3 (1.0)	1 (0.1)
Total	884	761	294	672
Received viral load test for newly diagnosed HIV positives*				
Yes	860 (92.8)	734 (95.7)	271 (90.9)	588 (82.3)
No	48 (5.2)	32 (4.2)	16 (5.4)	116 (16.3)
Unknown	19 (2.1)	1 (0.1)	11 (3.7)	10 (1.4)
Total	927 (100.0)	767 (100.0)	298 (100.0)	714 (100.0)
Referred to designated ART hospitals for newly diagnosed HIV positives*				
Yes	639 (68.9)	675 (88.0)	278 (93.3)	460 (64.4)
No	216 (23.3)	84 (11.0)	17 (5.7)	230 (32.2)
Unknown	72 (7.8)	8 (1.0)	3 (1.0)	24 (3.4)
Total	927 (100.0)	767 (100.0)	298 (100.0)	714 (100.0)
Received ART for newly diagnosed HIV positives*				
Yes	436 (68.2)	298 (44.2)	171 (61.5)	253 (55.0)
No	172 (26.9)	371 (55.0)	74 (26.6)	203 (44.1)
Unknown	31 (4.9)	6 (0.9)	33 (11.9)	4 (0.9)
Total	639 (100.0)	675 (100.0)	278 (100.0)	460 (100.0)
Days between WB confirmatory test and ART initiation (days)				
Mean ± SD^#^	12.0±20.1	19.6±29.2	3.1±10.6	16.0±27.8
<7	34 (7.8)	24 (8.1)	42 (24.6)	10 (4.0)
8–14	53 (12.2)	21 (7.1)	20 (11.7)	29 (11.5)
15–21	46 (10.6)	19 (6.4)	18 (10.5)	41 (16.2)
≥22	260 (59.6)	197 (66.1)	24 (14.0)	160 (63.2)
Unknown	43 (9.9)	37 (12.4)	67 (39.2)	13 (5.1)
Total	436	298	171	253

*Chi square test, p<0.001

^#^ Kruskal-Wallis equality-of-populations rank test, p<0.001

Regarding linkage to care and treatment (**Table 3**), Model D had the highest loss from screening to confirmatory testing among the four models with an 18.1% loss from first to second screening test and a further 5.9% loss from second screening to confirmatory testing. Model C showed the least attrition with only 1.3% loss between first screening and confirmatory tests. Model A and Model B showed similar levels of attrition. The positive predictive values (PPVs) of Model A for both the first rapid test (98.1%) and the second rapid test (98.8%) were comparable to Model B (97.4% and 98.5%, respectively; **Table 3**).

While Model B and Model C showed losses of only 0.8% and 1.3%, respectively, between diagnosis and receipt of CD4 cell count test, Model A and Model D had higher loss rates of 4.6% and 5.7%, respectively. Model B and Model C also showed relatively higher rates of referring HIV-positive MSM to ART hospitals (88.0% and 93.3%, respectively) over Model A and Model D (68.9% and 64.4%, respectively; χ^2^ = 158.9, p<0.001), while the rate of ART initiation was lowest in Model B compared with Models A, C and D (44.2% vs. 68.2%, 61.5% and 55.0%). However, when it came to ART initiation, Model C performed the best—52.8% (171/324) of cases that screened reactive (regardless of CD4 results) initiated ART, compared to Models A, B and D (38.9%, 436/1122; 34.2%, 298/870; and 21.1%, 253/1198; respectively, for an average of 30.9%). Thus, Model C was found to be 71% better at getting HIV-reactive MSM onto ART (**Table 3**).

Using Model A as a reference, multivariate logistic regression results showed the advantages of the Models B, C and D, which increased rates of CD4 cell count testing, referral to designated ART hospitals, and initiation of ART, when controlling for program city and other factors (**Table 4**). Regarding referring individuals screened reactive to confirmatory testing, no significant differences were observed between the four models after controlling for relevant factors (**Table 4**). Detailed results of multi-logistic analysis are provided in the supplementary materials (**S2–S5 Tables**).

**Table 4:** Logistic regression of four service delivery models on receiving WB confirmatory test and CD4 cell count test, referral to designated ART hospitals, and initiation of ART.

Models	Dependent Variables							
	Received WB confirmatory test (n = 3,089)		Received CD4 cell count test (n = 2,706)		Referred to designated ART Hospital (n = 2,706)		Initiated ART (n = 2,052)	
	OR	P-value	OR	P-value	OR	P-value	OR	P-value
**Model A**^a^	1.0		1.0		1.0		1.0	
**Model B**								
Unadjusted OR	1.77	<0.001	2.21	<0.001	2.78	<0.001	0.81	0.026
Adjusted OR^b^	0.75	0.489	7.02	0.004	3.45	<0.001	1.67	0.004
**Model C**								
Unadjusted OR	2.75	<0.001	2.54	<0.001	4.66	<0.001	1.75	<0.001
Adjusted OR^b^	1.59	0.510	55.54	<0.001	3.53	0.001	3.85	<0.001
**Model D**								
Unadjusted OR	0.27	<0.001	0.32	<0.001	0.46	<0.001	0.42	<0.001
Adjusted OR^b^	0.90	0.760	12.00	0.001	1.25	0.392	1.69	0.071

a:Reference group

b:Adjusted OR is based on multivariate logistic regressions controlling for socio-demographics, behavioral factors, screening test recruitment channels and screening test city.

The time differences between each step along the HIV continuum of care from the first screening test to ART initiation also varied by service delivery model (**Table 3**). Model D took an average of 3.3 days between the first and the second screening tests, while the other three models showed an average of less than one day (Kruskal-Wallis rank test, p<0.001). Model A took an average of 6.1 days between the second screening and the confirmatory testing, shorter than the other models (Kruskal-Wallis rank test, p<0.001). Between the confirmatory test and ART initiation, Model C was fastest at an average 3.1 days compared to the other models (Kruskal-Wallis rank test, p<0.001).

## Discussion

In this paper, we summarize key outcomes of this pilot model on increasing testing coverage, reducing follow-up losses and increasing ART uptake, in order to provide evidence to support future policy advocacy on HIV testing and ART promotion strategies for MSM with participation of CBOs. The success of a “Test and Treat” public health prevention approach depends on the effectiveness and efficiency of both case finding and case management [33]. The 2013 pilot study demonstrated that the key interventions adopted in this program—(a) empowering CBOs to conduct community-based rapid tests, and (b) streamlining testing and care procedures to minimize the number of patient-provider encounters and separate blood draws—can improve both effectiveness and efficiency of HIV interventions among MSM in China by reducing attrition along the HIV care continuum [12, 30, 33, 34].

Key indicators for testing and linkage to care improved substantially from 2012 to 2013, with a 35.8% increase in HIV tests provided, a 6% increase in the proportion of cases screened reactive receiving confirmatory tests, and a 65% reduction in loss to follow-up prior to CD4 cell count test. Although we did not collect information on ART initiation for newly-diagnosed cases in 2012, we believe it was very likely that ART initiation increased in the 2013 pilot. The reason for this is that in 2012, ART was mainly provided to those with CD4 counts less than 350 cells/mm^3^, whereas in 2013, only 20% of MSM who screened reactive had a CD4 this low.

The comparative analysis of four service delivery models provides further evidence supporting advantages of the applied interventions. With the participation of CBOs in provision of HIV testing and care (Model B, C, D), key indicators in linkage to care and treatment were significantly improved. The best service delivery model in the 2013 pilot was Model C, whereby CBOs conducted first rapid screening tests and referred MSM who screened reactive to designated ART hospitals for second rapid screening test. According to the multivariate analysis, this model showed significant improvements over the other three models in rates of CD4 testing, referral to designated ART hospitals, and initiation of ART. The particularly distinct advantages of designated ART hospitals over CDCs might be associated with the fact that CBOs, when working with the designated ART hospitals, were often located within the hospitals and worked closely with clinicians in a timely manner to manage risk of loss to follow-up. In these cases, CBO staff can easily and immediately deliver clients with reactive screening results to hospital clinicians for second rapid test. CBOs can also provide peer-based psychosocial support to newly-diagnosed MSM, which facilitates collection of correct contact information for future follow-up.

The advantage of the CBO-Hospital approach will become even more significant under the new policy China has adopted—providing ART to all people diagnosed with HIV infection regardless their CD4 results, as recommended by WHO [35, 36]. The designated ART hospitals in China will play a greater role in HIV case management under this new policy, as they treat all patients, not just late-stage of patients. Furthermore, early involvement of the designated ART hospitals also helps streamline referral procedures, eliminating risks of losses to follow-up associated with sending blood samples to city CDCs for CD4 and VL tests. Most designated ART hospitals are equipped with laboratories to conduct these tests and can do so more efficiently than city/district CDC laboratories.

As a cautionary note, the CBO-Hospital model relies heavily on a strong working relationship between ART hospitals and CBOs. Most hospitals in China’s current health system lack incentives to invest in HIV case management, let alone in working with CBOs. To realize this model, strong coordination by city public health authorities and dedicated financing for HIV case finding and case management are both needed to incentivize hospitals to realize their potential in HIV intervention. China’s current healthcare reforms might be a good opportunity to review hospital financing policies to encourage more hospitals to work on HIV case management and collaborate with CBOs [37].

The new process used in the 2013 pilot, a single post-screen clinic visit for blood draw, also greatly contributed to improvements in case follow up for confirmatory test, as well as CD4 cell count and viral load tests. The number of patient-provider encounters was reduced from three previously to just one. The proportion of cases screened reactive that received confirmatory tests and the proportion of newly-diagnosed HIV positive MSM receiving CD4 cell count tests in the 2013 pilot increased substantially when compared with 2012 for the same six cities. Several pilot studies carried out in China have demonstrated that simplified HIV testing and treatment procedures are helpful in shortening the time between testing and treatment, reducing loss to care and reducing mortality for AIDS patients.[38] Results of our streamlined model indicate that involvement of CBOs in testing, care and treatment may gain extra advantages. However, the fact that CD4 tests need to be performed within 48 hours after a blood sample was taken has raised concerns by local CDCs on possible waste of CD4 testing due to false positives. Our results showed the positive predictive value (PPV) of two rapid tests was 97.8%, suggesting wastage should not be a big concern. The timing of CD4 cell count testing did bring logistical challenges for county or district CDCs as CD4 cell count testing laboratory facilities are at city level. Equipping county/district CDCs to reduce the complicated logistical process is essential for the future.

Empowering CBOs to administer rapid HIV tests has been shown to be effective in scaling up testing and reducing follow-up losses [12, 33]. Moreover, the Consolidated Guideline for HIV Testing and Counselling issued by WHO in 2015 also recommends HIV testing by lay providers [37]. Results from our investigation provide further evidence in support this approach. We found that CBOs conducting both first and second rapid tests were as effective as CDCs conducting both rapid tests in terms of preventing loss to follow-up between first and second screen and between second screen and confirmatory testing. Thus, the positive predictive values (PPVs) for both first and second rapid tests done by CBOs are similar to that done by CDCs. This indicates that, after proper training, CBOs are capable of providing high-quality rapid testing services. These results should lessen concerns of local CDCs about the ability of CBOs to deliver quality HIV testing services. Moreover, the cases screened reactive by CBOs showed higher care and treatment retention rates than those cases screened reactive either by CDCs doing both first and second rapid tests or CBOs conducting first rapid tests and referring cases to CDCs for second screening tests. When CBOs were involved in both first and second rapid tests, they provided timely counseling to support clients in dealing with anxiety associated with HIV-reactive screening results and referred, and often accompanied, clients to local CDCs and hospitals for follow-up services. CBOs were not only helpful during the testing process, but also contributed to retaining MSM in care and treatment in the public health system [12]. When both rapid tests were conducted by CDCs (nearly 26% of cases screened reactive were tested by EIA), the HIV case management would not be initiated until the results of EIA were obtained, which usually took several days and required the client to return to CDCs on a separate visit. This may contribute to the relatively higher loss of cases screened reactive before receiving confirmatory tests. The model where CBOs conducted the first screening test and referred reactive individuals to CDCs for a second rapid test resulted in the highest follow-up losses among the four models. In both situations where CDCs conducted the second rapid tests (CDCs did both first and second rapid tests; CBOs did first and CDCs did the second rapid tests), CBOs easily participated in HIV case management.

Many local CDCs have legal and safety concerns about supporting CBOs in conducting rapid tests due to China’s current HIV testing policies and regulations allowing only approved medical institutions to provide HIV testing services [39]. However, the six cities in the 2013 pilot successfully adapted the program’s key interventions to their local situations, with only 14% of first rapid-test screening performed by CDCs (rather than CBOs as suggested by the program), and 25% of those screened HIV-reactive by CBOs were referred to CDCs for the second rapid-test screening (rather than CBOs as suggested by the program). CBOs referring MSM who screened reactive by first rapid tests to CDCs for second rapid tests occurred mostly in Beijing and Shanghai, where CBOs were advised by local CDCs to only use oral-based rapid tests due to CDC concerns about the biological safety risks associated with CBOs conducting finger-prick procedures. Our results showed that the CBO-CDC model did bring the highest loss (18.1%) of those screened reactive at the first test, with relatively long time periods between screening and confirmatory tests. Since 2015, the Chinese government has established an AIDS Fund with 50 million CNY each year specifically supporting CBOs in HIV prevention. Cooperating with CBOs in promoting HIV testing among MSM is one of its priorities. However, due to concerns around testing performed by CBOs, the CBO-CDC model is adopted by most areas. The scope of work at CBOs is still limited to HIV health education and behavioral intervention, and no clear description is given as to whether CBOs can provide rapid testing services for MSM. Our findings provide concrete evidence that CBOs bear significant advantages and should be further involved in HIV testing and case management.

It should be noted that the HIV prevalence among MSM screened by CBOs was significantly lower than that of MSM screened by CDCs. This may be because MSM tested by CBOs may have had a higher testing frequency than MSM tested by CDCs. Also MSM with higher risk behaviors may tend to choose CDCs for HIV testing, while MSM at sites where CBOs were offering screening may have taken the opportunity to have an HIV test even if they had a low risk of being HIV positive. Existing program data cannot explain such differences. However, the outcomes of the models in which CBOs supported HIV testing and care remained superior to other models despite the lower HIV prevalence among men screened. As a supplementary choice to the current HIV testing system, involvement of CBOs in HIV testing intervention and provision of testing services can significantly promote HIV testing and case finding for MSM, especially for those MSM who have concerns about privacy, confidentiality, and discrimination by health institutions.

### Limitations

Although this study introduced new interventions for program areas, it allowed program cities to adapt models based on their local situations. This makes the process complicated. The national health authorities have been advocating for the expansion of HIV testing and strengthening of HIV case management over the past several years. This may have produced positive bias for our program outcomes in 2013, especially when compared with 2012 results. We are also not able to evaluate the impact of some factors on the differences between the four screening (two-rapid tests) models. For example, the HIV rapid testing kits used in the first and second rapid tests differed between models, which may be associated with the rate of newly-diagnosed cases. Additionally, we did not include site/model specific characteristics, such as costs of tests or hours of service operation, which may account for some of the variation in testing and treatment outcomes by different models. Furthermore, although significant improvements were observed in the 2013 pilot, there is still significant attrition in the HIV care continuum. Based on informal communication with program implementation staff (at CBOs, CDCs, and hospitals in the six cities), the most frequent reasons for MSM not receiving second rapid tests and confirmatory testing were that they lived outside the city, were not located, or chose to go somewhere else for confirmation [40]. Further research is clearly needed in order to identify methods to further reduce loss of follow up. Finally, different implementation characteristics in the service delivery models between different CBOs and CDCs may limit the generalizability of the study findings.

## Conclusions

In a context whereby testing and treatment are key strategies for HIV prevention among MSM, case finding and management are critical. The present study has demonstrated that HIV rapid testing, involvement of CBOs, streamlining of testing and care procedures, and early hospital case management can improve testing, linkage to, and retention in, HIV care and treatment for MSM in China. We recommend these interventions to be adopted by China as national HIV prevention policy.

## Supporting Information

S1 Table Sample characteristics of MSM screened in 2013 (DOCX)

S2 Table Logistic regression analysis of receiving WB test on service delivery models (n = 3089) (DOCX)

S3 Table Logistic regression analysis of receiving CD4 test for newly identified HIV positives on service delivery models (n = 2706) (DOCX)

S4 Table Logistic regression analysis of referring newly identified HIV-positives to ART hospitals on service delivery models (n = 2706) (DOCX)

S5 Table Logistic regression analysis of initiating ART for newly identified HIV positives who were referred to ART hospitals on service delivery models (n = 2052) (DOCX)
