# Supplementary material for: Enhancing HIV Testing and Treatment among Men Who Have Sex with Men in China: A Pilot Model with Two-Rapid Tests, Single Blood Draw Session, and Intensified Case Management in Six Cities in 2013
Source: PLoS One. 2016 Dec 1;11(12):e0166812. doi: 10.1371/journal.pone.0166812 (PMC5131955; doi:10.1371/journal.pone.0166812)
Supplement: S1 Table — (DOCX) [file pone.0166812.s001.docx]

**S1 Table. Sample characteristics of MSM screened in 2013**

| **Variables** | **2013** | |
| --- | --- | --- |
|  | **N=72,577** | **%** |
| **Socio-demographic** |  |  |
| **Age** |  |  |
| ≤20 | 4,603 | 6.3 |
| 21-30 | 40,253 | 55.5 |
| 31-40 | 16,950 | 23.4 |
| 41-50 | 6,779 | 9.3 |
| ≥51 | 2,806 | 3.9 |
| Unknown | 1,186 | 1.6 |
| **Education** |  |  |
| High school attendance or less | 10,130 | 14.0 |
| Completed high school or vocational school | 21,592 | 29.8 |
| College attendance or higher | 40,544 | 55.9 |
| Unknown | 311 | 0.4 |
| **Marital status** |  |  |
| Single | 46,703 | 64.4 |
| Living with male partner | 5,545 | 7.6 |
| Married | 17,513 | 24.1 |
| Divorced or widowed | 2,267 | 3.1 |
| Unknown | 549 | 0.8 |
| **Current address** |  |  |
| In project city | 61,627 | 84.9 |
| Outside project city, within project province | 7,340 | 10.1 |
| Outside project province | 3,348 | 4.6 |
| Unknown | 262 | 0.4 |
| **Behavior** |  |  |
| **Ever had insertive anal sex** |  |  |
| Yes | 70,641 | 97.3 |
| No | 1,936 | 2.7 |
| **Had insertive anal sex in the past 6 months** (among ever had insertive anal sex) |  |  |
| Yes | 64,697 | 91.6 |
| No | 5,891 | 8.3 |
| Unknown | 53 | 0.1 |
| **Always used condoms during anal sex with males in past 3 months** (among had insertive anal sex in the past 6 months) |  |  |
| Yes | 42,886 | 66.3 |
| No | 21,531 | 33.3 |
| Unknown | 280 | 0.43 |
| **Ever tested for HIV** |  |  |
| Yes | 38,974 | 53.7 |
| No | 33,266 | 45.8 |
| Unknown | 337 | 0.5 |
| **HIV screening test** |  |  |
| **Screening test city** |  |  |
| Beijing | 26,111 | 36.0 |
| Chongqing | 7,923 | 10.9 |
| Nanjing | 9,474 | 13.1 |
| Shanghai | 9,483 | 13.1 |
| Wuhan | 10,392 | 14.3 |
| Xi'an | 9,194 | 12.7 |
| **Screening test recruitment channel** |  |  |
| Bar | 9,327 | 12.9 |
| Bathhouse | 7,716 | 10.6 |
| Park/public toilet | 15,110 | 20.8 |
| Internet | 21,350 | 29.4 |
| Others (mostly seeking testing on their own at CBOs offices) | 17,156 | 23.6 |
| Unknown | 1,918 | 2.6 |
| **Type of first HIV screening test** |  |  |
| Finger prick rapid tests | 43,368 | 59.8 |
| Oral based rapid tests | 29,038 | 40.0 |
| ELISA | 168 | 0.2 |
| Others | 3 | 0.0 |
| **First HIV screening test performed by** |  |  |
| CDC | 10,272 | 14.2 |
| CBO | 62,085 | 85.5 |
| Others | 220 | 0.3 |
| **Results of first HIV screening** |  |  |
| HIV(+) | 3,610 | 5.0 |
| HIV(-) | 68,967 | 95.0 |
| **Received second HIV screening test** (among positives of first screening tests) |  |  |
| Yes | 3,332 | 92.3 |
| No | 278 | 7.7 |
| **Second HIV screening test performed by** (among those received second HIV screening test) |  |  |
| CDC | 2,126 | 63.8 |
| CBO | 835 | 25.1 |
| Hospital | 330 | 9.9 |
| Unknown | 41 | 1.3 |
| **Type of second HIV screening test** (among those received second HIV screening test) |  |  |
| Finger prick rapid tests | 2,354 | 70.9 |
| Oral based rapid tests | 385 | 11.6 |
| ELISA | 579 | 17.4 |
| Others | 2 | 0.1 |
| Unknown | 0 | 0 |
| **Results of second HIV screening test** (among those received second HIV screening test) |  |  |
| HIV(+) | 3,180 | 95.4 |
| HIV(-) | 152 | 4.6 |
