# Supplementary material for: Enhancing HIV Testing and Treatment among Men Who Have Sex with Men in China: A Pilot Model with Two-Rapid Tests, Single Blood Draw Session, and Intensified Case Management in Six Cities in 2013
Source: PLoS One. 2016 Dec 1;11(12):e0166812. doi: 10.1371/journal.pone.0166812 (PMC5131955; doi:10.1371/journal.pone.0166812)
Supplement: S2 Table — (DOCX) [file pone.0166812.s002.docx]

**S2 Table.** **Logistic regression analysis of receiving WB test on service delivery models (n=3089)**

| Variables | B | S.E. | Wald | df | P-value | OR | 95% C.I. for OR | |
| --- | --- | --- | --- | --- | --- | --- | --- | --- |
|  |  |  |  |  |  |  | Lower | Upper |
| **Age** |  |  |  |  |  |  |  |  |
| <=30 |  |  | 8.870 | 2 | .012 |  |  |  |
| >=31 | .530 | .203 | 6.779 | 1 | .009 | 1.698 | 1.140 | 2.530 |
| Unknown | 1.109 | .616 | 3.245 | 1 | .072 | 3.031 | .907 | 10.129 |
| **Education** |  |  |  |  |  |  |  |  |
| High school attendance or less |  |  | 6.555 | 3 | .088 |  |  |  |
| Completed high school or vocational school | .253 | .261 | .940 | 1 | .332 | 1.288 | .772 | 2.146 |
| University attendance or higher | .593 | .248 | 5.735 | 1 | .017 | 1.810 | 1.114 | 2.942 |
| Unknown | 18.497 | 9917.558 | .000 | 1 | .999 | 107890766.063 | .000 | . |
| **Marriage** |  |  |  |  |  |  |  |  |
| Living with male partners |  |  | 3.929 | 4 | .416 |  |  |  |
| Single | .439 | .346 | 1.613 | 1 | .204 | 1.552 | .788 | 3.058 |
| Married | .055 | .384 | .021 | 1 | .885 | 1.057 | .498 | 2.241 |
| Divorced or widowed | .093 | .501 | .035 | 1 | .852 | 1.098 | .411 | 2.930 |
| Unknown | .972 | 1.115 | .760 | 1 | .383 | 2.643 | .297 | 23.500 |
| **City** |  |  |  |  |  |  |  |  |
| Beijing |  |  | 70.000 | 5 | .000 |  |  |  |
| Chongqing | 1.764 | .404 | 19.073 | 1 | .000 | 5.834 | 2.644 | 12.875 |
| Nanjing | 2.637 | .458 | 33.184 | 1 | .000 | 13.974 | 5.697 | 34.277 |
| Shanghai | 2.113 | .709 | 8.898 | 1 | .003 | 8.277 | 2.064 | 33.186 |
| Wuhan | -.161 | .400 | .163 | 1 | .687 | .851 | .388 | 1.865 |
| Xi'an | 2.454 | .610 | 16.166 | 1 | .000 | 11.635 | 3.518 | 38.488 |
| **No. of sexual partners** |  |  |  |  |  |  |  |  |
| <=1 |  |  | 1.624 | 2 | .444 |  |  |  |
| >=2 | .060 | .189 | .101 | 1 | .751 | 1.062 | .733 | 1.539 |
| Unknown | -.748 | .634 | 1.392 | 1 | .238 | .473 | .136 | 1.640 |
| **Condom use in the last month** |  |  |  |  |  |  |  |  |
| Never |  |  | 10.021 | 3 | .018 |  |  |  |
| Sometimes | -1.189 | .496 | 5.762 | 1 | .016 | .304 | .115 | .804 |
| Always | -.669 | .478 | 1.954 | 1 | .162 | .512 | .201 | 1.309 |
| Unknown | -.418 | .764 | .299 | 1 | .585 | .659 | .147 | 2.943 |
| **HIV test ever** |  |  |  |  |  |  |  |  |
| Yes |  |  | 9.311 | 2 | .010 |  |  |  |
| No | -.535 | .175 | 9.311 | 1 | .002 | .586 | .415 | .826 |
| Unknown | 18.902 | 11899.396 | .000 | 1 | .999 | 161887097.527 | .000 | . |
| **Recruitment channel** |  |  |  |  |  |  |  |  |
| Bar |  |  | 23.274 | 4 | .000 |  |  |  |
| Bath house | .158 | .360 | .194 | 1 | .659 | 1.172 | .579 | 2.371 |
| Park or public toilet | 1.328 | .507 | 6.852 | 1 | .009 | 3.772 | 1.396 | 10.195 |
| Internet | 1.139 | .295 | 14.917 | 1 | .000 | 3.124 | 1.752 | 5.568 |
| Others | .953 | .260 | 13.426 | 1 | .000 | 2.593 | 1.558 | 4.316 |
| **Model** |  |  |  |  |  |  |  |  |
| A: CDC+CDC |  |  | 1.408 | 3 | .704 |  |  |  |
| B: CBO+CBO | -.292 | .422 | .479 | 1 | .489 | .747 | .326 | 1.708 |
| C: CBO+HOSP | .464 | .703 | .435 | 1 | .510 | 1.590 | .401 | 6.306 |
| D: CBO+CDC | -.109 | .357 | .093 | 1 | .761 | .897 | .446 | 1.805 |
| **Constant** | 1.226 | .758 | 2.616 | 1 | .106 | 3.406 |  |  |
