# Supplementary material for: Enhancing HIV Testing and Treatment among Men Who Have Sex with Men in China: A Pilot Model with Two-Rapid Tests, Single Blood Draw Session, and Intensified Case Management in Six Cities in 2013
Source: PLoS One. 2016 Dec 1;11(12):e0166812. doi: 10.1371/journal.pone.0166812 (PMC5131955; doi:10.1371/journal.pone.0166812)
Supplement: S3 Table — (DOCX) [file pone.0166812.s003.docx]

**S3 Table. Logistic regression analysis of receiving CD4 test for newly identified HIV positives on service delivery models (n=2706)**

| Variables | B | S.E. | Wald | df | Sig. | OR | 95% C.I. for OR | |
| --- | --- | --- | --- | --- | --- | --- | --- | --- |
|  |  |  |  |  |  |  | Lower | Upper |
| **Age** |  |  |  |  |  |  |  |  |
| <=30 |  |  | 1.826 | 2 | .401 |  |  |  |
| >=31 | .162 | .263 | .381 | 1 | .537 | 1.176 | .702 | 1.971 |
| Unknown | -.566 | .535 | 1.123 | 1 | .289 | .568 | .199 | 1.618 |
| **Education** |  |  |  |  |  |  |  |  |
| High school attendance or less |  |  | 1.093 | 3 | .779 |  |  |  |
| Completed high school or vocational school | -.109 | .350 | .097 | 1 | .755 | .896 | .451 | 1.781 |
| University attendance or higher | .170 | .341 | .249 | 1 | .618 | 1.186 | .607 | 2.315 |
| Unknown | -.268 | 1.215 | .049 | 1 | .825 | .765 | .071 | 8.272 |
| **Marriage** |  |  |  |  |  |  |  |  |
| Living with male partners |  |  | 5.904 | 4 | .206 |  |  |  |
| Single | .387 | .450 | .739 | 1 | .390 | 1.473 | .609 | 3.561 |
| Married | .724 | .532 | 1.849 | 1 | .174 | 2.063 | .727 | 5.857 |
| Divorced or widowed | .246 | .668 | .136 | 1 | .713 | 1.279 | .345 | 4.738 |
| Unknown | -.917 | .828 | 1.225 | 1 | .268 | .400 | .079 | 2.028 |
| **City** |  |  |  |  |  |  |  |  |
| Beijing |  |  | 27.670 | 5 | .000 |  |  |  |
| Chongqing | 4.044 | .814 | 24.713 | 1 | .000 | 57.063 | 11.585 | 281.070 |
| Nanjing | 2.088 | .903 | 5.341 | 1 | .021 | 8.067 | 1.373 | 47.380 |
| Shanghai | 2.837 | .804 | 12.452 | 1 | .000 | 17.070 | 3.530 | 82.537 |
| Wuhan | 2.866 | .996 | 8.277 | 1 | .004 | 17.564 | 2.493 | 123.749 |
| Xi'an | 1.415 | .614 | 5.309 | 1 | .021 | 4.118 | 1.235 | 13.730 |
| **No. of sexual partners** |  |  |  |  |  |  |  |  |
| <=1 |  |  | 5.083 | 2 | .079 |  |  |  |
| >=2 | .597 | .271 | 4.835 | 1 | .028 | 1.816 | 1.067 | 3.090 |
| Unknown | .611 | .693 | .778 | 1 | .378 | 1.843 | .474 | 7.168 |
| **Condom use in the last month** |  |  |  |  |  |  |  |  |
| Never |  |  | 4.623 | 3 | .202 |  |  |  |
| Sometimes | .322 | .475 | .460 | 1 | .498 | 1.380 | .544 | 3.505 |
| Always | .335 | .379 | .783 | 1 | .376 | 1.398 | .666 | 2.936 |
| Unknown | -.968 | .704 | 1.894 | 1 | .169 | .380 | .096 | 1.508 |
| **HIV test ever** |  |  |  |  |  |  |  |  |
| Yes |  |  | .047 | 2 | .977 |  |  |  |
| No | -.050 | .240 | .043 | 1 | .835 | .951 | .594 | 1.524 |
| Unknown | -.089 | 1.262 | .005 | 1 | .944 | .915 | .077 | 10.841 |
| **Recruitment channel** |  |  |  |  |  |  |  |  |
| Bar |  |  | 33.560 | 4 | .000 |  |  |  |
| Bath house | 1.524 | .670 | 5.172 | 1 | .023 | 4.591 | 1.234 | 17.077 |
| Park or public toilet | .451 | .405 | 1.243 | 1 | .265 | 1.570 | .710 | 3.472 |
| Internet | 1.615 | .384 | 17.719 | 1 | .000 | 5.029 | 2.371 | 10.667 |
| Others | 1.802 | .367 | 24.119 | 1 | .000 | 6.060 | 2.952 | 12.437 |
| **Model** |  |  |  |  |  |  |  |  |
| A: CDC+CDC |  |  | 21.433 | 3 | .000 |  |  |  |
| B: CBO+CBO | 1.949 | .670 | 8.447 | 1 | .004 | 7.019 | 1.886 | 26.117 |
| C: CBO+HOSP | 4.017 | .900 | 19.912 | 1 | .000 | 55.544 | 9.514 | 324.286 |
| D: CBO+CDC | 2.485 | .727 | 11.696 | 1 | .001 | 11.999 | 2.889 | 49.846 |
| **Constant** | -2.216 | 1.046 | 4.491 | 1 | .034 | .109 |  |  |
