# Supplementary material for: Enhancing HIV Testing and Treatment among Men Who Have Sex with Men in China: A Pilot Model with Two-Rapid Tests, Single Blood Draw Session, and Intensified Case Management in Six Cities in 2013
Source: PLoS One. 2016 Dec 1;11(12):e0166812. doi: 10.1371/journal.pone.0166812 (PMC5131955; doi:10.1371/journal.pone.0166812)
Supplement: S4 Table — (DOCX) [file pone.0166812.s004.docx]

**S4 Table. Logistic regression analysis of referring newly identified HIV positives to ART hospitals on service delivery models (n=2706)**

| Variables | B | S.E. | Wald | df | Sig. | OR | 95% C.I. for OR | |
| --- | --- | --- | --- | --- | --- | --- | --- | --- |
|  |  |  |  |  |  |  | Lower | Upper |
| **Age** |  |  |  |  |  |  |  |  |
| <=30 |  |  | 1.106 | 2 | .575 |  |  |  |
| >=31 | .031 | .128 | .059 | 1 | .808 | 1.032 | .802 | 1.326 |
| Unknown | -.281 | .292 | .924 | 1 | .336 | .755 | .426 | 1.339 |
| **Education** |  |  |  |  |  |  |  |  |
| High school attendance or less |  |  | 4.442 | 3 | .217 |  |  |  |
| Completed high school or vocational school | -.010 | .178 | .003 | 1 | .955 | .990 | .699 | 1.403 |
| University attendance or higher | .182 | .170 | 1.138 | 1 | .286 | 1.199 | .859 | 1.675 |
| Unknown | -1.015 | .797 | 1.622 | 1 | .203 | .362 | .076 | 1.728 |
| **Marriage** |  |  |  |  |  |  |  |  |
| Living with male partners |  |  | 15.287 | 4 | .004 |  |  |  |
| Single | -.359 | .285 | 1.586 | 1 | .208 | .698 | .399 | 1.221 |
| Married | .243 | .313 | .603 | 1 | .437 | 1.275 | .691 | 2.354 |
| Divorced or widowed | -.202 | .371 | .296 | 1 | .586 | .817 | .395 | 1.691 |
| Unknown | .650 | .653 | .991 | 1 | .320 | 1.916 | .533 | 6.895 |
| **City** |  |  |  |  |  |  |  |  |
| Beijing |  |  | 313.847 | 5 | .000 |  |  |  |
| Chongqing | .032 | .310 | .011 | 1 | .917 | 1.033 | .563 | 1.895 |
| Nanjing | 1.155 | .584 | 3.914 | 1 | .048 | 3.174 | 1.011 | 9.964 |
| Shanghai | -1.381 | .315 | 19.235 | 1 | .000 | .251 | .136 | .466 |
| Wuhan | -.375 | .368 | 1.036 | 1 | .309 | .688 | .334 | 1.415 |
| Xi'an | -3.754 | .262 | 205.687 | 1 | .000 | .023 | .014 | .039 |
| **No. of sexual partners** |  |  |  |  |  |  |  |  |
| <=1 |  |  | 3.896 | 2 | .143 |  |  |  |
| >=2 | .205 | .120 | 2.922 | 1 | .087 | 1.228 | .970 | 1.554 |
| Unknown | .606 | .512 | 1.398 | 1 | .237 | 1.833 | .671 | 5.002 |
| **Condom use in the last month** |  |  |  |  |  |  |  |  |
| Never |  |  | 4.293 | 3 | .232 |  |  |  |
| Sometimes | .411 | .242 | 2.887 | 1 | .089 | 1.509 | .939 | 2.424 |
| Always | .270 | .196 | 1.900 | 1 | .168 | 1.310 | .892 | 1.924 |
| Unknown | -.340 | .530 | .412 | 1 | .521 | .712 | .252 | 2.012 |
| **HIV test ever** |  |  |  |  |  |  |  |  |
| Yes |  |  | .662 | 2 | .718 |  |  |  |
| No | .044 | .119 | .136 | 1 | .712 | 1.045 | .828 | 1.319 |
| Unknown | .611 | .824 | .551 | 1 | .458 | 1.843 | .367 | 9.263 |
| **Recruitment channel** |  |  |  |  |  |  |  |  |
| Bar |  |  | 54.416 | 4 | .000 |  |  |  |
| Bath house | 1.275 | .344 | 13.712 | 1 | .000 | 3.577 | 1.822 | 7.023 |
| Park or public toilet | -.472 | .247 | 3.641 | 1 | .056 | .624 | .384 | 1.013 |
| Internet | .435 | .199 | 4.762 | 1 | .029 | 1.545 | 1.045 | 2.284 |
| Others | .925 | .203 | 20.699 | 1 | .000 | 2.522 | 1.693 | 3.756 |
| **Model** |  |  |  |  |  |  |  |  |
| A: CDC+CDC |  |  | 43.353 | 3 | .000 |  |  |  |
| B: CBO+CBO | 1.234 | .230 | 28.784 | 1 | .000 | 3.436 | 2.189 | 5.394 |
| C: CBO+HOSP | 1.260 | .386 | 10.651 | 1 | .001 | 3.525 | 1.654 | 7.512 |
| D: CBO+CDC | .222 | .259 | .732 | 1 | .392 | 1.248 | .751 | 2.076 |
| **Constant** | .694 | .503 | 1.903 | 1 | .168 | 2.002 |  |  |
