# Supplementary material for: Enhancing HIV Testing and Treatment among Men Who Have Sex with Men in China: A Pilot Model with Two-Rapid Tests, Single Blood Draw Session, and Intensified Case Management in Six Cities in 2013
Source: PLoS One. 2016 Dec 1;11(12):e0166812. doi: 10.1371/journal.pone.0166812 (PMC5131955; doi:10.1371/journal.pone.0166812)
Supplement: S5 Table — (DOCX) [file pone.0166812.s005.docx]

**S5 Table. Logistic regression analysis of initiating ART for newly identified HIV positives who were referred to ART hospitals on service delivery models (n=2052)**

| Variables | B | S.E. | Wald | df | Sig. | OR | 95% C.I. for OR | |
| --- | --- | --- | --- | --- | --- | --- | --- | --- |
|  |  |  |  |  |  |  | Lower | Upper |
| **Age** |  |  |  |  |  |  |  |  |
| <=30 |  |  | 12.795 | 2 | .002 |  |  |  |
| >=31 | .356 | .120 | 8.775 | 1 | .003 | 1.428 | 1.128 | 1.808 |
| Unknown | .958 | .410 | 5.466 | 1 | .019 | 2.606 | 1.168 | 5.817 |
| **Education** |  |  |  |  |  |  |  |  |
| High school attendance or less |  |  | 8.113 | 3 | .044 |  |  |  |
| Completed high school or vocational school | .050 | .154 | .106 | 1 | .745 | 1.051 | .777 | 1.422 |
| University attendance or higher | .334 | .151 | 4.917 | 1 | .027 | 1.397 | 1.040 | 1.876 |
| Unknown | 1.056 | .963 | 1.202 | 1 | .273 | 2.876 | .435 | 19.008 |
| **Marriage** |  |  |  |  |  |  |  |  |
| Living with male partners |  |  | 6.645 | 4 | .156 |  |  |  |
| Single | -.245 | .225 | 1.188 | 1 | .276 | .783 | .504 | 1.216 |
| Married | .136 | .251 | .294 | 1 | .587 | 1.146 | .700 | 1.876 |
| Divorced or widowed | -.019 | .320 | .003 | 1 | .954 | .982 | .524 | 1.839 |
| Unknown | -.033 | .518 | .004 | 1 | .949 | .967 | .350 | 2.672 |
| **City** |  |  |  |  |  |  |  |  |
| Beijing |  |  | 155.088 | 5 | .000 |  |  |  |
| Chongqing | .550 | .317 | 2.999 | 1 | .083 | 1.732 | .930 | 3.227 |
| Nanjing | 3.098 | .541 | 32.783 | 1 | .000 | 22.148 | 7.670 | 63.953 |
| Shanghai | 2.658 | .357 | 55.422 | 1 | .000 | 14.267 | 7.087 | 28.724 |
| Wuhan | -.304 | .356 | .731 | 1 | .392 | .738 | .367 | 1.482 |
| Xi'an | 1.799 | .429 | 17.573 | 1 | .000 | 6.046 | 2.607 | 14.022 |
| **No. of sexual partners** |  |  |  |  |  |  |  |  |
| <=1 |  |  | 3.625 | 2 | .163 |  |  |  |
| >=2 | .180 | .119 | 2.301 | 1 | .129 | 1.197 | .949 | 1.511 |
| Unknown | -.465 | .499 | .870 | 1 | .351 | .628 | .236 | 1.669 |
| **Condom use in the last month** |  |  |  |  |  |  |  |  |
| Never |  |  | 5.703 | 3 | .127 |  |  |  |
| Sometimes | -.460 | .238 | 3.726 | 1 | .054 | .632 | .396 | 1.007 |
| Always | -.367 | .185 | 3.948 | 1 | .047 | .693 | .483 | .995 |
| Unknown | .192 | .512 | .140 | 1 | .708 | 1.211 | .444 | 3.305 |
| **HIV test ever** |  |  |  |  |  |  |  |  |
| Yes |  |  | .039 | 2 | .981 |  |  |  |
| No | -.019 | .112 | .028 | 1 | .866 | .981 | .788 | 1.222 |
| Unknown | -.093 | .866 | .012 | 1 | .914 | .911 | .167 | 4.975 |
| **Recruitment channel** |  |  |  |  |  |  |  |  |
| Bar |  |  | 23.224 | 4 | .000 |  |  |  |
| Bath house | .217 | .260 | .695 | 1 | .405 | 1.242 | .746 | 2.066 |
| Park or public toilet | .898 | .241 | 13.853 | 1 | .000 | 2.455 | 1.530 | 3.939 |
| Internet | .763 | .182 | 17.597 | 1 | .000 | 2.145 | 1.502 | 3.064 |
| Others | .524 | .191 | 7.544 | 1 | .006 | 1.688 | 1.162 | 2.453 |
| **Model** |  |  |  |  |  |  |  |  |
| A: CDC+CDC |  |  | 28.272 | 3 | .000 |  |  |  |
| B: CBO+CBO | .515 | .177 | 8.513 | 1 | .004 | 1.674 | 1.184 | 2.366 |
| C: CBO+HOSP | 1.348 | .345 | 15.287 | 1 | .000 | 3.849 | 1.959 | 7.566 |
| D: CBO+CDC | .523 | .289 | 3.265 | 1 | .071 | 1.687 | .957 | 2.973 |
| **Constant** | -1.593 | .476 | 11.189 | 1 | .001 | .203 |  |  |
